# Multidisciplinary expert consensus on secondary fracture prevention in Spain

**DOI:** 10.1007/s11657-021-00878-w

**Published:** 2021-02-27

**Authors:** E. Casado, J. Blanch, C. Carbonell, J. C. Bastida, J. L. Pérez-Castrillón, L. Canals, L. Lizán

**Affiliations:** 1 Servicio de Reumatología, Hospital Universitari Parc Taulí (UAB), Sabadell, Spain; 2 Servicio de Reumatología, Hospital del Mar, Barcelona, Spain; 3 Instituto Catalán de la Salud (ICS), Barcelona, Spain; 4 Universidad de Barcelona, Barcelona, Spain; 5 Medicina de familia, centro de Salud de Marín, Pontevedra, Spain; 6 Coordinador nacional Grupo de Osteoporosis SEMG, Pontevedra, Spain; 7 Servicio Medicina Interna, Hospital Universitario Rio Hortega, Valladolid, Spain; 8 Amgen (Europe) GmBH, Rotkreuz, Switzerland; 9 Outcomes’10, Castellón, Spain; 10 Departamento de Medicina, Universidad Jaime I, Castellón, Spain

**Keywords:** Fragility, Fracture, Secondary prevention, Osteoporosis, Delphi, Consensus

## Abstract

**Summary:**

The study aimed to achieve expert consensus to optimize secondary fracture prevention in Spain. Relevant gaps in current patient management were identified. However, some aspects were considered difficult to apply. Future efforts should focus on those items with greatest divergences between importance and feasibility.

**Purpose:**

To establish a Spanish multidisciplinary expert consensus on secondary fracture prevention.

**Methods:**

A two-round Delphi consensus was conducted, guided by a Scientific Committee. The 43-item study questionnaire was designed from a literature review and a subsequent multidisciplinary expert group (*n* = 12) discussion. The first-round questionnaire, using a 7-point Likert scale, assessed the experts’ *opinion* of the current situation, their *wish* for items to happen, and their *prognosis* that items would be implemented within 5 years. Items for which consensus was not achieved were included in the second round. Consensus was defined as ≥ 75% agreement or ≥ 75% disagreement. A total of 102 experts from 14 scientific societies were invited to participate.

**Results:**

A total of 75 (response rate 73.5%) and 69 (92.0%) experts answered the first and second Delphi rounds, respectively. Participants mean age was 51.8 years [standard deviation (SD): 10.1 years]; being 24.0% rheumatologists, 21.3% primary care physicians, 14.7% geriatricians, 8.0% internal medicine specialists, 8.0% rehabilitation physicians, and 8.0% gynecologists. Consensus was achieved for 79.1% of items (wish, 100%; prognosis, 58.1%). Effective secondary prevention strategies identified as requiring improvement included: clinical report standardization, effective hospital primary care communication (telephone/mail and case managers), health-related quality of life (HRQoL) questionnaires use, and treatment compliance monitoring (prognosis agreement 33.3%, 47.8%, 18.8%, and 55.1%, respectively).

**Conclusion:**

A consensus was reached by health professionals in their wish to implement strategies to optimize secondary fracture prevention; however, they considered some difficult to apply. Efforts should focus on those items with currently low application and those with greatest divergence between wish and prognosis.

## Introduction

Population aging is boosting osteoporosis (OP) and fragility fracture incidence [1]. These fractures represent a high burden, being associated with significant morbidity, mortality, and healthcare costs [1, 2]. Following major fractures, almost half of patients, particularly the elderly, never regain previous function; one-third requiring important changes to their living environments; and hip fractures are associated with a two- to three-fold increase in mortality [2]. Additionally, OP diagnosis may lead to psychological and physical consequences, impacting patients’ health-related quality of life (HRQoL) [3–7].

Patients with a record of fragility fracture are at a higher risk of new fractures compared to the general population [8–10]. Therefore, most guidelines advise OP treatment to reduce the number of new fractures in this cohort [11].

Numerous studies have clearly documented an important treatment gap after a fragility fracture [1]. Although OP is a chronic condition requiring a long-term management plan [12], initiation of OP therapy lags behind other disease areas, with a decrease in bisphosphonate prescription in recent years in Europe and the USA [2, 13, 14]. Less than 20% of individuals suffering a fragility fracture receive treatment within the following year to reduce the risk of future fractures [15]. In Spain, only 5–8% of patients with hip fracture received prior bone protection medication, while at discharge, this percentage represents 21–37%, and one-month post-fracture, it is 38–41% [16, 17]. Furthermore, patients at high risk of fractures who are prescribed OP drugs frequently withdraw from treatment within the first year [12]. In Spain, 50% of patients register poor compliance or discontinue OP treatment during the first year [18]. As a result, there is an important gap between patients who would benefit from treatment and those who actually receive it [13].

Several scientific organizations have called for an improvement in fragility fracture management in order to prevent subsequent fractures and restore functional abilities and life quality [1]. Thus, it is time for a “call to action” to the professionals involved in fragility fracture management.

Our study aims to reach a consensus among multidisciplinary experts on the best measures required to optimize fragility fracture management of patients in Spain.

## Methods

A two-round Delphi was conducted. The Delphi method is a systematic approach to obtain opinions of a group of experts by means of a series of short self-administered questionnaires [19].

### Participants

To create the multidisciplinary Delphi panel of experts, the main Spanish societies involved in fragility fracture patient management and other osteoporosis-related associations (*n* = 14) participated in the study: Spanish Association for the Study of Menopause (AEEM); Hispanic Foundation for Osteoporosis and Metabolic Diseases (FHOEMO); Spanish Society of Healthcare Executives (SEDISA); Spanish Society of Endocrinology and Nutrition (SEEN); Spanish Society of Osteoporotic Fractures (SEFRAOS); Spanish Society of Geriatric Medicine and Gerontology (SEGG); Spanish Society for Bone and Mineral Metabolism Research (SEIOMM); Spanish Society of Geriatric Medicine (SEMEG); Spanish Society of Primary Health Care Physicians (SEMERGEN); Spanish Society of Family and Community Medicine (SEMFYC); Spanish Society of General and Family Medicine (SEMG); Spanish Society of Internal Medicine (SEMI); Spanish Society of Rheumatology (SER); and Spanish Society of Rehabilitation and Physical Medicine (SERMEF).

These societies were responsible for selecting the participants among their members. Selected participants were physicians who practiced a medical specialty related to each specific society, with expertise in osteoporotic fracture management. Every expert received an e-mail describing the purpose of the study and a web link to the survey.

### Scientific committee

The project was led by a scientific committee comprising five experts in fragility fractures (two rheumatologists [SEIOMM], two general practitioners [SEMERGEN, SEMG], and one internal medicine physician [SEMI]). These members were selected by the promoter of the study (SEIOMM) among the different members of the societies collaborating in the study, considering their expertise in fragility fractures and a multidisciplinary composition of the committee.

### Design

The study questionnaire was designed based on a literature review on secondary fracture prevention and a multidisciplinary expert discussion group (general practitioners [*n* = 2]; specialists in rheumatology [*n* = 2]; gynecology [*n* = 1], internal medicine [*n* = 1]; geriatrics [*n* = 2], physical therapy [*n*= 1]; endocrinology [*n* = 1]; pharmacists [*n* = 1]; healthcare managers [*n* = 1]) and overseen by the Scientific Steering Committee. First-round questionnaire was available from March 6 to April 7, 2019, while the second-round was available from May 13 to June 6, 2019.

The first-round questionnaire included sociodemographic variables and 20 questions, comprising a total of 43 items exploring different aspects of secondary fracture prevention in Spain. In each question posed, the experts were asked to express their opinion from the *current* perspective as well as their *wish* and their *prognosis* regarding the future (5-year period). Each question was scored in a 7-point Likert scale (1, strongly disagree; 2, mostly disagree; 3, somewhat disagree; 4, neither agree nor disagree; 5, somewhat agree; 6, mostly agree; 7, strongly agree), corresponding to the extent to which the expert agreed with the item being addressed. Participants were also provided with a free-text space in which they could make observations and comments.

The second-round questionnaire comprised the questions for which no consensus was reached in the first round. Only the *wish* and *prognosis* perspectives were included, since the *current* perspective described the present situation and did not require consensus. This second questionnaire was tailored specifically for each expert. Every question contained information regarding the score he/she recorded in the first round and the position of the overall group (range of the greatest percentage of scores). Each expert was invited to confirm his/her position or modify the score to bring it closer to that of the group, so that a consensus could be reached on the greatest possible number of questions asked.

### Data analysis

For each question, consensus was achieved when at least 75% of participants reached agreement (5, somewhat agree; 6, mostly agree; or 7, strongly agree) or disagreement (1, strongly disagree; 2, mostly disagree; or 3, somewhat disagree). The former was referred to as “agreement consensus”, as the majority of experts wanted it to happen or predicted it would happen, whereas the latter was called “disagreement consensus”, since most experts did not want it to happen or predicted it would not happen.

The strategies proposed with the lowest application (*current*, ≥ 75.0% disagreement), requiring implementation (*wish*, ≥ 75.0% agreement) but unlikely to be applied (prognosis ˂ 75.0% agreement), were identified as those strategies requiring main attention. For these strategies, consensus was analyzed for the whole of participants but also according to medical specialty (considering the specialties with > 10.0% of participation in the study) and time of professional experience (above or below average time of experience).

## Results

### Participants

Of the 102 experts invited to participate in the study, 75 fully answered the first-round questionnaire (response rate 73.5%), while 69 experts fully answered the second-round questionnaire (response rate 92.0%).

Participants average age was 51.8 years [standard deviation (SD): 10.1 years], 60.0% men and 40.0% women, with professional experience of 22.3 years [SD: 10.9 years]. Expert distribution by medical specialties was as follows: rheumatology 24.0% (*n* = 18), primary care (PC) 21.3% (*n* = 16), geriatrics and gerontology 14.7% (*n* = 11), internal medicine 8.0% (*n* = 6), physical medicine and rehabilitation 8.0% (*n* = 6), obstetrics and gynecology 8.0% (*n* = 6), endocrinology and nutrition 6.7% (*n* = 5), orthopedic surgery and traumatology 4.0% (*n* = 3), health executive 2.7% (*n* = 2), and others 2.7% (*n* = 2) (Table 1).

**Table 1:** Sociodemographic variables of participants

Variable	Value
Age [mean (SD)]	51.8 (10.1)
Sex [%(*N*)]	
Women	40.0% (30)
Men	60.0% (45)
Scientific society*	
SEIOMM: Spanish Society for Research on Bone and Mineral Metabolism	57.3% (43)
SER: Spanish Society of Rheumatology	25.3% (19)
SEFRAOS: Spanish Society of Osteoporotic Fractures	17.3% (13)
SEMERGEN: Spanish Society of Primary Health Care Physicians	14.7% (11)
SEMEG: Spanish Society of Geriatric Medicine	12.0% (9)
SEGG: Spanish Society of Geriatric Medicine and Gerontology	10.7% (8)
SEMG: Spanish Society of General and Family Medicine	10.7% (8)
AEEM: Spanish Association for the Study of Menopause	10.7% (8)
FHOEMO: Hispanic Foundation for Osteoporosis and Metabolic Diseases	9.3% (7)
SERMEF: Spanish Society of Rehabilitation and Physical Medicine	9.3% (7)
SEMI: Spanish Society of Internal Medicine	8.0% (6)
SEDISA: Spanish Society of Healthcare Executives	6.7% (5)
SEEN: Spanish Society of Endocrinology and Nutrition	6.7% (5)
Others	9.3% (7)
Medical specialty [%(*N*)]	
Rheumatology	24.0% (18)
Primary care	21.3% (16)
Geriatrics and gerontology	14.7% (11)
Internal medicine	8.0% (6)
Physical medicine and rehabilitation	8.0% (6)
Obstetrics and gynecology	8.0% (6)
Endocrinology and nutrition	6.7% (5)
Orthopedic surgery and traumatology	4.0% (3)
Health executive	2.7% (2)
Others	2.7% (2)
Mean time of professional experience, years (SD)	22.3 (10.9)
Hospital type [%(*N*)]^	
Group 1: < 200 beds	10.6% (7)
Group 2: 200–500 beds	31,8% (21)
Group 3: 501–1000 beds	25.8% (17)
Group 4: > 1000 beds	30.3% (20)
Not applicable	1.5% (1)
Specific resources for fracture patients at the hospital [%(*N*)]^	
Fracture liaison service (FLS)	33.3% (22)
Others	25.8% (16)
Orthogeriatric unit	13.6% (9)
Specific osteoporosis or bone metabolism unit	4.5% (3)
Close cooperation with orthopedic surgery and traumatology service	3.0% (2)
Fracture unit	1.5% (1)
FLS in development	1.5% (1)
None	36.7% (18)
Not applicable	3.0% (2)

*Participants could be members of more than one scientific society

^Questions answered by 66 out of 75 participants

### Current situation and consensus (wish and prognosis)

Consensus was reached on 79.1% of items (wish, 100%; prognosis, 58.1%) (Table 2).

**Table 2:** Results of the Delphi consultation: current, wish and prognosis perspectives

Question	Current (%)*			Wish (%)			Prognosis (%)		
	**D**	**I**	**A**	**D**	**I**	**A**	**D**	**I**	**A**
1. Specific educational campaigns are carried out for patients who have suffered a fragility fracture	64.0	4.0	32.0	2.7	1.3	**96.0**	11.6	5.8	**82.6**
2. The following agents and/or groups are involved in awareness and educational campaigns regarding the prevention of fragility fractures:									
2.1. Pharmaceutical industry 2.2. Scientific societies 2.3. Healthcare professionals 2.4. Patient advocacy groups 2.5. Media 2.6. Health managers	6.7 13.3 38.7 30.7 37.3 72.0	2.7 6.7 14.7 22.7 20.0 18.7	90.7 80.0 46.7 46.7 42.7 9.3	1.3 0 1.3 4.0 6.7 6.7	1.3 0 1.3 5.3 2.7 1.3	**97.3** **100** **97.3** **90.7** **90.7** **92.0**	4.0 2.7 12.0 15.9 14.5 62.3	0 4.0 8.0 11.6 14.5 14.5	**96.0** **93.3** **80.0** 72.5 71.0 23.2
3. There are specific health policies for the improvement of secondary prevention of fragility fractures:									
3.1 At the national level 3.2 At the regional level	56.0 58.7	12.0 9.3	32.0 32.0	1.3 4.0	6.7 4.0	**92.0** **92.0**	18.8 47.8	15.9 8.7	65.2 43.5
4. The following strategies are applied to improve care and/or compliance in the secondary prevention of fragility fractures									
4.1 Promotion of healthy lifestyle and fall prevention 4.2 Treatment prescription optimization 4.3. Implementation of fracture liaison services (FLS) or specific resources for fracture patients 4.4 Patient information/training 4.5 Promotion patient-physician communication 4.6 Continuous training of healthcare personnel 4.7 Promotion of social/family support	33.3 45.3 38.7 57.3 49.3 48.0 66.7	17.3 17.3 12.0 12.0 18.7 9.3 16.0	49.3 37.3 49.3 30.7 32.0 42.7 17.3	2.7 2.7 4.0 2.7 1.3 5.3 2.7	4.0 4.0 2.7 1.3 8.0 0 6.7	**93.3** **93.3** **93.3** **96.0** **90.7** **94.7** **90.7**	5.8 7.2 5.8 5.8 11.6 11.6 31.9	7.2 11.6 14.5 17.4 14.5 15.9 24.6	**87.0** **81.2** **79.7** **76.8** 73.9 72.5 43.5
5. The implementation of specific resources for the patient with fragility fracture (such as FLS) allows for better control and follow-up of patients and for resources savings for the health system by reducing the number of new fragility fractures.	26.7	13.3	60.0	2.7	1.3	**96.0**	4.3	5.8	**89.9**
6. The different clinical practice guidelines define standardized criteria for secondary prevention of fragility fractures.	25.3	4.0	70.7	0	2.7	**97.3**	4.0	10.7	**85.3**
7. To prevent a new fracture, all patients with fragility fracture receive:									
7.1 Recommendations on healthy measures and habits 7.2 Active pharmacological treatment for osteoporosis 7.3 Recommendations on Calcium and Vitamin D Supplements 7.4 Rehabilitation and fall prevention programs	48.0 72.0 33.3 66.7	16.0 6.7 17.3 9.3	36.0 21.3 49.3 24.0	2.7 2.7 4.0 2.7	1.3 1.3 2.7 4.0	**96.0** **96.0** **93.3** **93.3**	8.7 15.9 9.3 15.9	5.8 5.8 13.3 14.5	**85.5** **78.3** **77.3** 69.6
8. The "treat-to-target" strategy is applied in the secondary prevention of fragility fractures	73.3	14.7	12.0	10.7	10.7	**78.7**	31.9	14.5	53.6
9. When discussing treatment for secondary fracture prevention, the patient receives clear and concise information from the primary care physician or hospital:									
9.1 The importance of therapeutic compliance 9.2 Fracture status and prognosis 9.3 The risk-benefit balance of the therapeutic options	56.0 54.7 61.3	13.3 22.7 14.7	30.7 22.7 24.0	2.7 1.3 1.3	0 2.7 4.0	**97.3** **96.0** **94.7**	8.7 11.6 8.7	8.7 11.6 18.8	**82.6** **76.8** 72.5
10. In routine clinical practice, a systematic control of treatment compliance is performed for the secondary prevention of fragility fractures**	80.0	6.7	13.3	2.7	1.3	**96.0**	34.8	10.1	55.1
11. Systematic recording of fragility fractures in the patient's medical record allows better identification and secondary prevention of fragility fractures	28.0	12.0	60.0	1.3	2.7	**96.0**	0	5.8	**94.2**
12. There is a registry of fragility fractures:									
12.1 At the national level 12.2 At the regional level	42.7 65.3	12.0 12.0	45.3 22.7	2.7 4.0	4.0 5.3	**93.3** **90.7**	14.5 37.7	7.2 13.0	**78.3** 49.3
13. There is an efficient communication system between hospital and primary care that allows effective follow-up of the fracture patient, through									
13.1 Shared health record 13.2 Telephone and/or specific e-mail address between assistance levels** 13.3 Case Manager (specific liaison person between primary care and hospital)**	38.7 78.7 84.0	6.7 8.0 8.0	54.7 13.3 8.0	1.3 9.3 1.3	4.0 5.3 6.7	**94.7** **85.3** **92.0**	8.0 31.9 37.7	12.0 20.3 14.5	**80.0** 47.8 47.8
14. After a fragility fracture, the hospital doctor sends a report to the primary care doctor proposing the most appropriate specific treatment.	65.3	8.0	26.7	2.7	2.7	**94.7**	8.7	13.0	**78.3**
15. The clinical reports of the fracture patient sent from hospital to primary care are standardized at the hospital level**	86.7	5.3	8.0	2.7	4.0	**93.3**	52.2	14.5	33.3
16. There are protocols for the treatment of the patient with fragility fracture, in the case of:									
16.1 Hip fracture (specific protocol) 16.2 Fragility fractures in general 16.3 Vertebral fracture (specific protocol) 16.4 Other fractures (specific protocols for humerus, wrist...)	25.3 37.3 41.3 58.7	5.3 10.7 12.0 21.3	69.3 52.0 46.7 20.0	1.3 2.7 1.3 1.3	4.0 1.3 4.0 5.3	**94.7** **96.0** **94.7** **93.3**	9.3 10.1 10.1 17.4	4.0 7.2 10.1 15.9	**86.7** **82.6** **79.7** 66.7
17. In long-term follow-up of the patient, there is an established protocol about "drug holidays" (drug treatment rest periods) of bisphosphonates and restoration of medication in treatment for secondary prevention of fragility fractures.	54.7	10.7	34.7	5.3	2.7	**92.0**	13.0	10.1	**76.8**
18. In making decisions about treatment for secondary fracture prevention, the health professional considers the patient's preferences	40.0	9.3	50.7	1.3	6.7	**92.0**	10.1	7.2	**82.6**
19. To promote patient involvement, there are specific tools (patient-directed informational material, in paper or on-line format) to assist decision-making on treatment for secondary prevention of fragile fractures	52.0	14.7	33.3	5.3	6.7	**88.0**	5.8	13.0	**81.2**
20. In clinical practice, questionnaires are used to assess the health-related quality of life (HRQoL) of the patient with fragility fracture**	85.3	5.3	9.3	6.7	12.0	**81.3**	65.2	15.9	18.8

*D* disagreement, *I* indifference, *A* agreement

Wish (desire for it to happen); prognosis (belief that it will happen in a five-year period)

*Current perspective was not subject to formal consensus

**Strategies requiring main attention

#### Education and specific policies

There are no specific educational campaigns for patients or health policies aimed specifically at improving secondary fracture prevention in Spain. However, most participants (wish, 96.0%; prognosis, 82.6%) agreed that educational campaigns for patients with fractures should be implemented and would also like to have specific health policies (wish, 92.0% both at national and regional levels), but this latter would be unlikely to happen mid-term (agreement 65.2% at national and 43.5% at regional level, respectively).

The main agents involved in educational campaigns are the pharmaceutical industry and scientific societies, while healthcare managers are the least involved, according to participants. Participants agreed that all agents should be involved in educational campaigns, considering that the pharmaceutical industry (prognosis, 96.0%), scientific societies (prognosis, 93.3%), and health professionals (prognosis, 80.0%) should be involved. However, mid-term involvement of patient associations, media, and healthcare managers was deemed to be less probable.

#### Strategies to improve care/compliance

Application of the seven strategies proposed (promotion of healthy lifestyle and fall prevention, treatment prescription optimization, implementation of fracture liaison services (FLS) or specific resources for fracture patients, patient information/training, promotion patient-physician communication, continuous training of healthcare personnel, promotion of social/family support) to improve patient care and/or treatment compliance in secondary fracture prevention varies. Patient information or social support promotion is the least common. Although participants agreed that all the strategies should be put into practice to improve fracture patient care, only the promotion of healthy lifestyle and prevention of falls (prognosis, 87.0%), OP treatment optimization (prognosis, 81.2), FLS or other specific strategies implementation (prognosis, 79.7%), and patient information (prognosis, 76.8%) were considered feasible for mid-term implementation.

Experts considered that the implementation of specific resources for fracture patients, such as FLS, contributes to patient control and health resource saving and agreed it improves patient follow-up (wish, 96.0%; prognosis, 89.9%).

#### Approaches to prevent fractures

Most experts considered that the criteria for secondary fracture prevention in clinical guidelines are standardized (wish, 97.3%; prognosis, 85.3%). However, currently, not all patients with fragility fracture are optimally managed to prevent new fractures. Participants considered the provision of OP treatment both necessary and feasible (wish, 96.0%; prognosis, 78.3%), recommendations on calcium and vitamin D intake (wish, 93.3%; prognosis, 77.3%) or healthy lifestyle promotion (wish, 96.0%; prognosis, 85.5%), while a specific rehabilitation program for preventing falls was found to be difficult to implement (wish, 93.3%; prognosis, 69.6%). A “treat-to-target” strategy is not currently applied, and although it was regarded as important (wish, 78.7%), experts considered that the situation would not change in the short term (prognosis, 53.6%).

At present, health professionals do not provide appropriate information to the patient about the state and prognosis of the fracture, the risk-benefit of therapeutic options, nor the importance of treatment compliance. Participants agreed that this information should be provided to the patient though fewer thought the benefit-risk balance of treatment options would be implemented mid-term (prognosis, 72.5%).

Finally, OP treatment compliance is not currently assessed in a systematic fashion, and despite its relevance (wish, 96.0%), participants believed it would remain so (prognosis, 55.1%).

#### Fracture identification

Experts considered that fracture coding in medical history contributes to the prevention of new fractures, since it facilitates identification. In accordance with the current situation, participants agreed (wish, 96.0%; prognosis, 94.2%) that better secondary fracture prevention could be achieved by systematically recording fragility fractures in the clinical chart.

National or regional fracture registries are not widespread in Spain. However, while participants considered that national registries should and would be established (wish, 93.3%; prognosis, 78.3%), they perceived that regional registries would be implemented less (prognosis, 49.3%).

#### Hospital-primary care communication

In general, no effective communication systems between the hospital and PC exist for patient follow-up. Although the participants agreed that the three strategies proposed (specific telephone/e-mail, case manager and shared health record) should be used, they considered that only communication through shared health records was feasible (prognosis, 80.0%).

The content of the fracture patient’s clinical report is not standardized among hospitals, and it is not referred to PC. Participants agreed (wish, 94.7%; prognosis, 78.3%) that a clinical report, including treatment recommendation, should and would be submitted to PC at patient discharge. However, it would not be feasible to standardize the minimum measures to be implemented mid-term (prognosis, 33.3%).

#### Treatment protocols

Experts considered that treatment protocols are not widespread in Spain. Accordingly, although participants reflected there should be established protocols for all fracture profiles, protocols for the treatment of other fractures (humerus, wrist, etc.) would not be developed mid-term (prognosis, 66.7%).

“Drug holidays” related to bisphosphonate treatment are not generally addressed by existing protocols; nonetheless, participants considered they would be in the future (wish, 92.0%; prognosis, 76.8%).

#### Patient perspective

Only half of the experts expressed that treatment-related decision-making considers patient preferences and that patient decision aids are available for a shared decision-making. On the other hand, patient HRQoL is not assessed in current clinical practice. Participants agreed that patient preferences would be taken into greater consideration in the future (wish, 92.0%; prognosis, 82.6%) and decision aids to promote patient involvement would become available mid-term (wish, 88.0%; prognosis, 81.2%). The use **of HRQoL questionnaires** was considered necessary (wish, 81.3%), but not likely in the near future (prognosis, 18.8%).

### Strategies requiring main attention

The consensus reached for the whole of participants regarding the four strategies requiring main attention (Table 2) is maintained (*current*: ≥ 75.0% disagreement, *wish*: ≥ 75.0% agreement, *prognosis*: no consensus) when analyzed by medical specialty (rheumatology vs. PC vs. geriatrics and gerontology) and time of professional experience (≤> 22.3 vs. > 22.3 years) (data not shown).

## Discussion

### Main findings

In this Delphi survey, a multidisciplinary group of experts in the field of osteoporotic fractures agreed on the measures to be adopted to optimize the management of patients with fragility fractures. The findings of our study indicate the changes required to improve secondary fracture prevention in routine clinical practice. Likewise, these results provide key information to help stakeholders anticipate future hurdles and unmet needs in the management of patients with fragility fractures in Spain.

Participants agreed on the need to apply all the strategies proposed (wish), which highlights health professionals’ interest in improving current secondary fracture prevention strategies. However, regarding their prognosis of future implementation, consensus was only reached for 58.1% of questions. This means that although experts consider the proposed strategies are necessary, around 40% are deemed unlikely to be implemented.

To overcome the current barriers for effective secondary fracture prevention, attention should be paid to the following strategies, which are currently the least implemented and whose implementation is considered unlikely mid-term: (1) standardization of the clinical reports of fracture patients discharged from hospital; (2) implementation of effective communication channels between hospital and primary care, such as a specific telephone/e-mail or a case manager, for patient follow-up; (3) use of HRQoL questionnaires to consider patient perspective in treatment decision-making; and (4) systematic control of OP treatment compliance.

Other recommendable strategies with currently low application are the involvement of healthcare managers in educational campaigns, the development of specific healthcare policies for fracture prevention, the promotion of social support initiatives to improve patient care, and the establishment of national fracture registries. Finally, the following are also considered relevant: the implementation of fall prevention programs, the provision of patient information about the risk-benefit of the therapeutic options, the existence of protocols for the treatment of non-hip-non-vertebral fractures, and the implementation of a treat-to-target strategy to optimize treatment.

### Results in the context of existing literature

In 2016, leading researchers and bone health experts from different foundations met at the ASBMR Annual Meeting and issued a call to action with recommendations to assertively address the critical care gap and reduce fracture risk in our aging population [20]. Since this meeting, many other health organizations have joined, urging the case for promoting the prevention of second and subsequent fractures in patients who have sustained their first fragility fracture [21].

In response to this call to action, our study has identified the main issues that should be addressed in Spain to optimize fracture prevention. Changes in clinical practice should be performed to address the current management gaps identified, and efforts should focus on those items reflecting the greatest divergence between importance (wish) and feasibility (prognosis). In agreement with our results, previous studies have highlighted the need to optimize coordination between primary and specialized care [22, 23], which becomes important after an osteoporotic fracture has been repaired [24].

Currently, the patient-centered approach to decision-making is a hot topic, and this has also arisen as a major issue in our study. The care community should gain a better understanding of the patient’s fragility fracture experience so that direct improvements in care can be based on the perspectives of end users [25]. As a consequence of this gap, there is little social awareness of OP and fragility fractures [26], and many patients do not understand the long-term significance and importance of their fracture [27]. Thus, there is a major need to improve patient information about the disease and the importance of treatment, with the aim of improving long-term OP treatment compliance [26]. In this regard, several drugs have demonstrated to be safe and effective in reducing the risk of fractures [28], so there is a major opportunity to involve patients in decision-making to find a treatment option matching their preferences (e.g., administration mode). A systematic control of treatment compliance was also found to be an important factor in our study. This agrees with the patients’ perspective, as women with OP perceive that the condition should be taken seriously and strategies for remembering to take medication are necessary [29]. Interventions targeting osteoporotic fracture prevention that encourage collaboration between patients and healthcare professionals could incorporate approaches for shared decision-making or other similar approaches to achieve medication compliance and patient education. Shared decision-making is reported as the most effective therapeutic option to increase medical treatment compliance and persistence, as well as fostering greater patient satisfaction and improving the healthcare processes and outcomes for patients [30].

In line to improve fracture patient care and compliance experts considered, the implementation of specific resources for fracture patients, such as FLS, is feasible in mid-term. FLS are care-coordinator-based secondary fracture prevention programs that systematically identify fragility fracture patients and treat them [31]. Their implementation contributes to an effective communication between the hospital and PC and ensures continuity of care [26, 32]. Several studies indicated that they are a cost-effective strategy for reducing the OP care gap, refracture rate, and mortality [33, 34].

According to expert opinion, the study also pinpointed the need for better communication with patients regarding fracture risk and fracture risk reduction, the benefits and risks of receiving treatment or not, and the importance of a healthy lifestyle [32]. A multisector effort is required to support patients and their clinicians in undertaking meaningful discussions on these issues [26]. This point is highly relevant, given that the lack of communication between healthcare providers and patients is frequently identified as a key barrier to proper OP management and secondary fracture prevention [32].

With regard to prioritization of fragility fracture prevention in national policy, there is much to be done [26]. Successful transformation of care is a matter of healthcare policy and also relies upon consensus among all participants in the multidisciplinary team that cares for fragility fracture patients [35]. Therefore, the recommendations provided are intended not only to help clinicians in patient management but also assist policy-makers in the design and implementation of strategies and pave the way for future research. In this respect, there is a need to create the necessary structure and to provide resources so that the issue becomes a matter of the health system rather than of the physician. Specific polices should be made to help achieve the goals of this call to action, with the participation of all the stakeholders involved.

Fall prevention is another currently neglected issue [1], which the study participants considered unfeasible to implement mid-term, and therefore, it should be addressed in clinical practice. Many patients suffering a fall, even if uninjured, develop a fear of falling and tend to limit their activity, resulting in reduced mobility, and loss of bone mass and physical fitness [36]. The COVID-19 pandemic has undoubtedly had, and will continue to have, a significant impact on the lives of people living with OP. Social distancing and self-isolation are likely to be resulting in changes in activity levels (and consequently may impact rates of falls and fractures) [37]. Fall prevention needs comprehensive management, including nutrition, prescription medicine, changes in lifestyle, and exercise schedules, among others [36].

Finally, if healthcare providers and decision-makers are unaware of a patient’s fracture record and diagnosis with OP, they cannot take steps to provide the long-term care required for this lifelong, chronic condition [27]. Therefore, experts point out that systematically recording fragility fractures in the clinical chart facilitates patient identification and contributes to the prevention of new fractures. Specific fracture registries are needed at both the national and local (regional) levels. The Spanish National Hip Fracture Registry is a continuous registry of patients admitted for a hip fracture in a large group of Spanish hospitals, and recent publications suggest that it is gaining momentum [38, 39]. For instance, in the UK, clinical data for each patient are captured and stored in Clinical Practice Research Datalink (CPRD), one of the world’s largest databases of primary care electronic health records [40], including reliable recording of fracture events [41].

The WHO has designated the period 2020–2030 as the Decade of Healthy Aging. If the mobility and independence of older people are to be maintained as a new demographic era develops, a determined effort must be made to fulfill many of the “wishes” identified.

### Implications for practice and further research

The agreed strategies to optimize fracture prevention could be of value not only for helping clinicians to manage their patients but also for OP-related researchers, and for policy-makers to implement changes in current clinical practice in Spain. Endorsement of the proposed strategies within national healthcare policies and advocacy programs can achieve alignment of the objectives of professionals, policy-makers, and patients [35].

Additionally, the results of our study contribute to anticipating future needs and providing a basis for further discussions on how to achieve secondary fracture prevention. Other countries could benefit from replicating this approach.

### Strengths and limitations of the study

An important aspect of the Delphi technique is the choice of an appropriate panel of participants. In this study, our aim was to obtain a sample representing all medical specialties (and their respective scientific societies) involved in the management of fragility fractures. Therefore, the multidisciplinary composition of the Delphi panel, together with their vast professional experience, is one of the main strengths of the study, as it brings a wide range of expertise and experience to the decision-making process. It is of great value that healthcare executives and professionals from different specialties and scientific societies have reached a common consensus and thus reflect unmet needs in OP management. Regarding the number of participants in our Delphi study, we consider it to be sufficient given a minimum of 10–18 panel members have been suggested by other authors [42].

Another strength lies in the fact that the questionnaire was drawn up under the guidance of a scientific committee and the expert discussion group, helping to define the appropriate and inappropriate approaches to current gaps in care. Consequently, the questions posed were relevant to participants and facilitated a consensus regarding the professionals’ wishes. Of note, some of the strategies identified may refer to unmet needs related to the health system and thus could apply to different health conditions. Nonetheless, their specific relevance in secondary fracture prevention was established in this study.

One limitation is that the study was performed in Spain; therefore, the results should be interpreted in their context and may not be applicable to other settings. It would be interesting to undertake a similar study using the Delphi methodology with international experts and to compare the results.

Another limitation is the arbitrary consensus cut-off. We decided to set the level of consensus at ≥ 75%, a threshold frequently reported in the literature. The use of another threshold could give rise to different results.

## Conclusion

In this Delphi survey, a multidisciplinary group of experts from 14 different societies reached a consensus on strategies that could be implemented to improve secondary fracture prevention in Spain. Efforts should focus on those items with currently low application and for which there is greatest divergence between participants’ wishes and prognosis. Accordingly, the main items requiring improvement are the standardization of clinical reports of fracture patients, efficient communication between hospitals and PCs for patient follow-up, the use of HRQoL questionnaires to consider the patient's perspective, and the systematic monitoring of OP treatment compliance. The results of this study represent the first step to optimizing secondary fracture prevention in Spain in the future.
